# Pharyngoesophageal diverticulum mimicking thyroid nodules: Some interesting ultrasonographic signs

**DOI:** 10.3389/fonc.2023.1030014

**Published:** 2023-02-07

**Authors:** Zhiqun Bai, Xuemei Wang, Zhen Zhang

**Affiliations:** Department of Ultrasonic Diagnosis, The First Hospital of China Medical University, Shenyang, Liaoning, China

**Keywords:** pharyngoesophageal diverticulum, Zenker’s diverticulum, Killian–Jamieson diverticulum, thyroid nodules, ultrasonographic features

## Abstract

**Objective:**

To analyze the ultrasonographic features of pharyngoesophageal diverticulum (PED) mimicking thyroid nodules and to explore the clinical value of ultrasonography in the diagnosis of PED.

**Method:**

The sonographic findings of 68 patients with PED were retrospectively reviewed. According to the diverticulum echo intensity characteristics, the lesions were divided into solid nodular diverticulum, gas-containing nodular diverticulum, liquid-containing nodular diverticulum, and atypical diverticular changes; and the ultrasonographic manifestations were compared among the four groups.

**Results:**

30/68 were solid nodular diverticula. The diverticulum cavity was oval or elliptic with a clear border, and the diverticulum wall suggested exhibited a typical hyper-hypo-hyper-echogenic pattern. The diverticulum wall and esophageal wall were seen to be continuous if multiple sections were scanned, and hypoechoic walls showed punctate blood flow. 29/68 diagnosed with air-containing nodular diverticulum, lesions appeared with gas-like hyper-echogenicity internally, with some amount of gas and change in the tail pattern during swallowing. 6/68 patients were diagnosed with liquid-containing nodular diverticulum, and the main ultrasonic manifestations were an anechoic internal diverticulum cavity that was clearly bounded from the thyroid but continuous with the esophageal wall, with a typical hyper-hypo-echoless pattern from the outside to the inside. Another 3/68 were found to have atypical diverticular changes, regional convexities of the esophageal wall with unfashioned nodules. The convex segment was continuous with the hyper-hypo-echogenic esophageal wall and could be seen on slitting scanning.

**Conclusion:**

Overall, PEDs mimicking thyroid nodules have specific ultrasonographic features. Familiarity with them can avoid missed diagnoses and misdiagnoses.

## Introduction

Pharyngoesophageal diverticulum (PED) is a rare benign disease of the esophagus in and is always on the left side. However, it can sometimes mimic thyroid disease, leading to misdiagnosis (1, 2). Zenker’s diverticulum (ZD) is the most common type of PED and was first described by Friedrich Albert von Zenker, a German pathologist, in 1867 (3). ZD occurs in the posterior wall of the esophagus due to a defect between the ring pharynx and stenosis of the pharyngeal muscles, which causes the esophagus to burst behind the mucous membranes (3). Killian–Jamieson diverticulum is a rare esophageal diverticulum that occurs at a ratio of 1:4 to ZD, with a prevalence rate between 0.0025% and 0.025%; it is characterized by an evagination of a muscular gap in the anterolateral of the wall of the esophagus (4). Both types of PED can simulate thyroid nodules, and physical examination, ultrasonography, and computed tomography may lead to clinicians unaware of PED’s specific imaging features to mistake PED for thyroid nodules (5),resulting in unnecessary ultrasound-guided fine needle aspiration (FNA) or surgical treatment (6).

However, while PED is a benign disease of the esophagus, esophago-fiberscopes and transesophageal echocardiography probes may mistakenly enter the diverticulum cavity, especially during ultrasound-guided FNA, which can penetrate the thin diverticulum wall, leading to subsequent inflammation, infection, and other clinical symptoms (7). Therefore, it is important to differentiate between PED and thyroid nodules. We collected and collated 68 patients with PED to analyze images of the lesion and identify sonographic diagnostic features.

## Materials and methods

### Research subjects

Retrospective analyses were performed on 68 patients who were suspected to have PED at the First Affiliated Hospital of China Medical University from June 2011 to June 2021. Among them, 63 patients were confirmed to have PED following a barium swallow test. Ultrasonographic findings in 5 patients had typical signs of air, and there was movement relative to the thyroid during swallowing. Diverticulum and esophageal wall that were continuous when multiple sections were scanned on at least two follow-ups were considered separate PEDs. The patients’ ages ranged 18–82 years (mean, 41.7 ± 3.1 years), and 44 were women. 61 cases were misdiagnosed as thyroid nodules or parathyroid nodules, and the remaining seven cases were detected incidentally during thyroid sonography.

There were no special clinical symptoms in 62 patients; 4 patients inadvertently touched the front-neck block and moved it during swallowing, and 2 patients clinically showed swelling in the front of the neck and had difficulty swallowing.

The inclusion criteria were as follows: 1) PED with a well-established ultrasound examination and confirmed using barium meal examination or ultrasound follow-up; and 2) a “nodule” with a close relationship to the thyroid gland that may be misdiagnosed as a thyroid nodule.

The exclusion criteria were as follows: 1) PED located outside the anatomical location of the thyroid gland; and 2) patients with suspected PED on ultrasonography only but refused further examination to confirm the diagnosis.

All methods were performed in accordance with the guidelines set forth in the Declaration of Helsinki. All patients provided oral or written informed consent to participate in the study before biopsy.

### Inspection methods

#### Ultrasonography

The diagnostic instruments used were a Preirus^®^ (linear array probe = 5-12 MHz; Hitachi, Tokyo, Japan), AixPlorer^®^ (linear array probe = 4-15 MHz; Supersonic, France), and IU22^®^ (linear array probe = 5-12 MHz; Philips, Amsterdam, Netherlands).

With patients in the supine position, thyroid ultrasonography was performed routinely to determine the internal echo, with or without thyroid nodules. Thyroid image reporting and data system (TI-RADS) grading was performed (8). When cervical nodules were detected at the level of the thyroid, the size, echogenicity, shape, border, blood flow, and location of the lesion were recorded and stored for future review.

To determine whether the lesion was connected to the adjacent esophageal wall, changes in the lesion shape, echogenicity, and movement relative to the thyroid were observed during the patient’s ingestion of water. To consider if a barium meal examination was appropriate, gastroscopy or follow-up observation was recommended.

Thyroid function tests, such as thyroglobulin level, antithyroid autoantibody to thyroglobulin ratio, and thyroid microsomal antigen level, were performed using a Losi cobas e601 immunoassay analyzer (Roche Diagnostics Indiana, USA).

#### X-ray barium meal examination

An Aquillion radiographic image enhancement system (Toshiba, Tokyo, Japan) with an IBS system was used. Standard X-ray barium meal examination was performed at the Department of Radiology of the First Affiliated Hospital of China Medical Hospital. After 4 h of fasting, the patients received resuspended barium sulfate. The patients were assessed in a standard posture. Images were obtained in the right front oblique position, left front oblique position, and positive position. The movement of the barium sulfate suspension in the esophagus was examined to identify whether there was bag-like extrusion or its limitations increased in size.

### Classification of the PED

**Figure 1:**
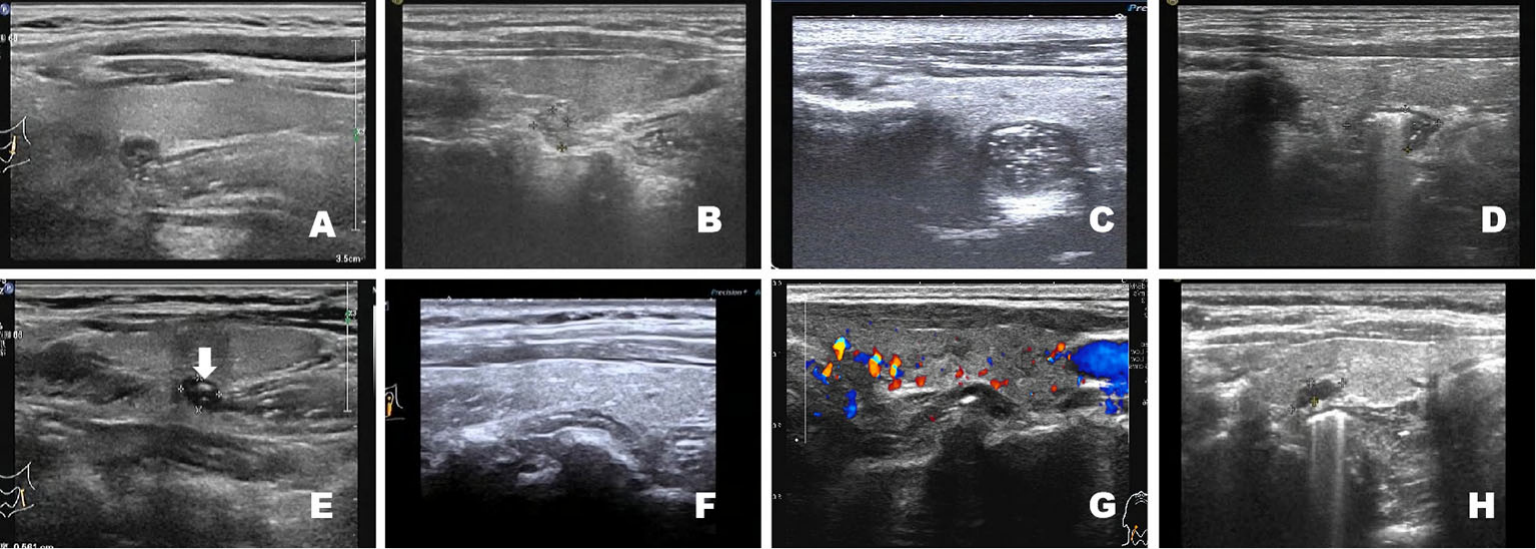
Compared with the echogenicity of the thyroid gland, solid nodular diverticula are classified as solid hypoechoic nodular diverticula **(A)**, image from a 29-year-old woman), solid isoechoic nodular diverticula **(B)**, image from a 56-year-old man), and solid high-low echogenic intermixed nodular diverticula **(C)**, image from a 34-year-old woman). Air-containing nodular diverticulum. The lesion contains a central strongly echogenic area associated with a comet-tail artifact. The presence of gas can be seen, and the tail pattern changed during swallowing **(D)**, image from a 32-year-old man). Liquid-containing nodular diverticulum with echoless interior **(E)**, white arrow, image from a 46-year-old man). Atypical diverticular changes: Regional convexities of the esophageal wall with no obvious nodules **(F)**, image from a 64-year-old man; **(G)**, image from a 51-year-old man; **(H)**, image from a 53-year-old woman).

According to the internal echo, there were four types of ultrasonic features of PED in our study:

(1) Solid nodular diverticulum: The lesion was an oval-shaped mixed nodule, with outpouching of the esophageal wall and almost located on the left lobe of the thyroid gland. Compared to the echogenicity of the thyroid gland, this type is divided into solid hypoechoic nodular diverticula, solid isoechoic nodular diverticula, and solid high-low echogenic intermixed nodular diverticula (**Figures 1A–C**).
The nodules may project into the thyroid parenchyma, thus making it more difficult to distinguish from a thyroid nodule, or they can be located behind the tegument at the posterior margin of the thyroid. The diverticulum is typically full of food debris or esophageal mucosal folds, with few or no air echoes.
(2) Air-containing nodular diverticulum: The lesion contained a central strong echogenic area associated with a comet-tail artifact, and the amount of gas and the tail pattern changed during swallowing (**Figure 1D**).
(3) Liquid-containing nodular diverticulum: The lesion was echoless on internal dynamic scanning or after drinking water; the internal echoless parts were visible on flow sensing. There was movement relative to the thyroid during swallowing actions (**Figure 1E**).
(4) Atypical diverticular changes: Regional convexities of the esophageal wall with no obvious nodules (**Figures 1F–H**).

### Statistical analysis

We used SPSS v23.0 (IBM, Armonk, NY, USA). Measurement data are presented as the mean ± SD. Student’s t-test was used to compare the maximum diameter of the “T” and “V” acoustic tails in the air-containing nodular diverticulum group. Statistical significance was set at *p*<0.05.

## Results

### Clinical and pathology results

**Figure 2:**
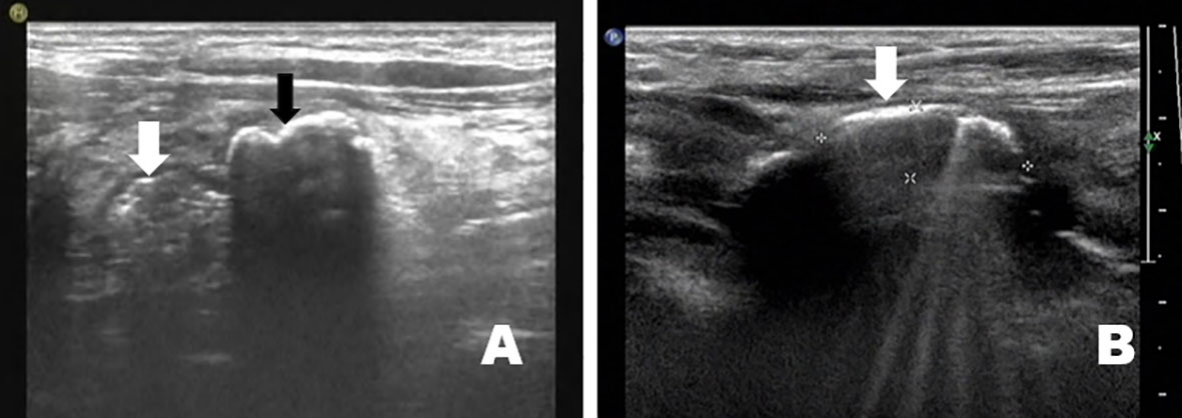
PED was found in combination with thyroid nodules in 73.5% (50/68) of cases **(A)**, image from a 56-year-old man). After thyroidectomy, a PED appeared in the thyroid region **(B)**, image from a 42-year-old man).

A total of 68 patients were included in the study. In two patients, the diverticulum was located behind the right lobe of the thyroid gland, while in 66 it was located behind the left lobe. Eight patients had Hashimoto’s thyroiditis, four had autoimmune thyroiditis, three had hyperthyroidism, and 50 had combined thyroid nodules (TI-RADS 3, n=32; TI-RADS 4a, n=15; TI-RADS 4b, n=2; TI-RADS 4c, n=1) (**Figure 2A**), as well as three cases after thyroidectomy (**Figure 2B**). Immunoreactive parathyroid hormone (IPTH) and serum calcium and phosphorus levels were within the normal range in all patients. The sonographic findings and diagnostic features of PED are presented in **Table 1**.

**Table 1:** Physical and laboratory examinations.

	Solid nodular diverticulum	Air-contained nodular diverticulum	Liquid-containing nodular diverticulum	Atypical diverticular alters
Age (years)				
≥45	21	18	2	2
<45	9	11	4	1
Sex				
Male	12	11	1	1
Female	18	18	5	2
Long diameter (mm)	17.7 ± 12.1	11.2 ± 5.3	12.1 ± 4.7	16.4 ± 11
Location				
The right	6	4	1	0
The left	24	25	5	3
With thyroid nodules				
Yes	26	20	2	2
No	4	9	4	1
Serum calcium				
Normal	27	27	3	3
Abnormal	3	2	3	0
thyroid hormone and related antibody				
Normal	24	27	2	2
Abnormal	6	2	4	1
Diagnostic method				
Barium meal test	27	26	6	3
Follow-up	3	3	0	0

### Ultrasonographic findings

#### Solid nodular diverticulum

**Figure 3:**
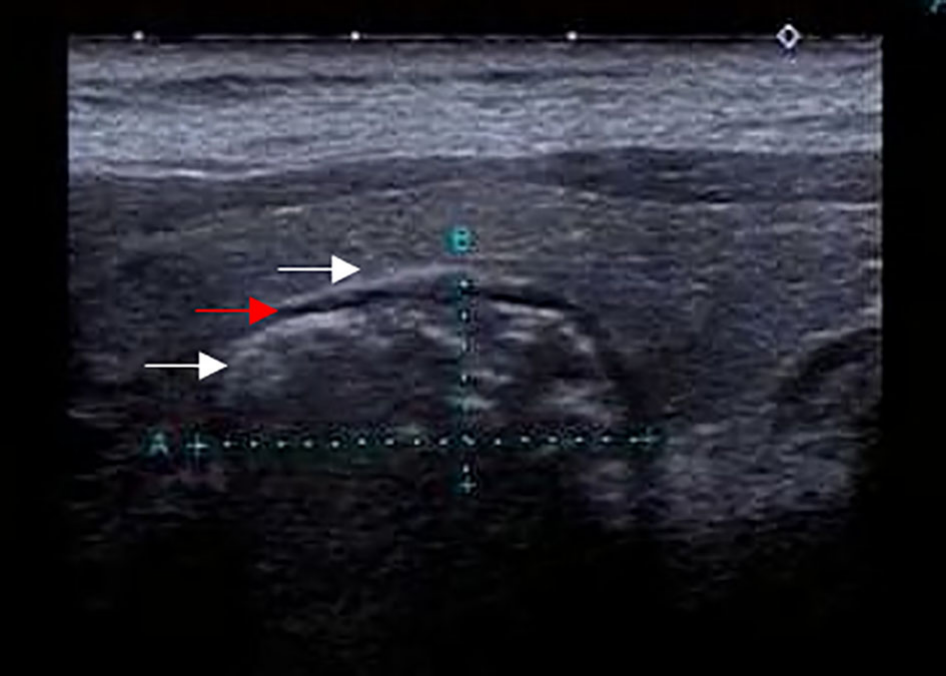
The solid nodular diverticulum wall has a typical hyper-hypo-hyper echogenic pattern (image from a 37-year-old woman).

**Figure 4:**
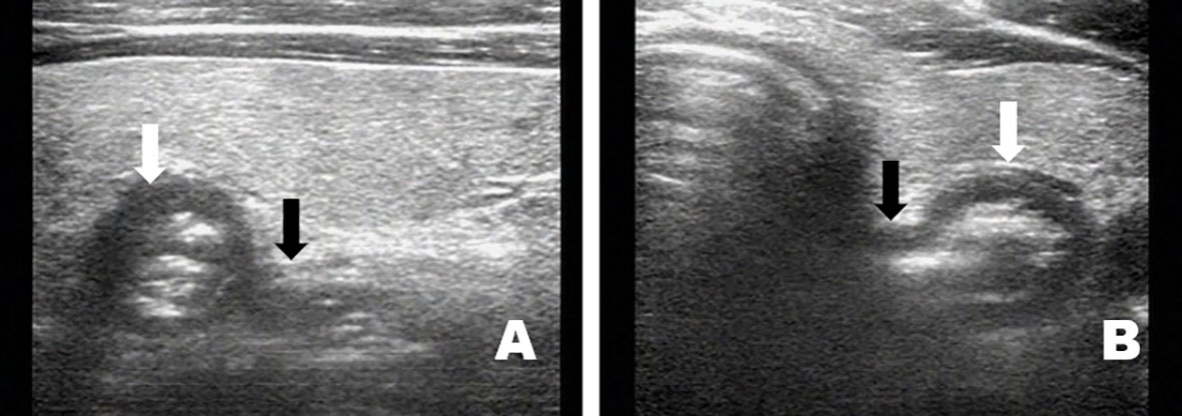
The solid nodular diverticulum wall and esophageal wall are continuous if viewed with multiple sections such as crosscut and then cut scanned **(A)**, image from a 21-year-old man: **(B)**, image from a 39-year-old man).

**Figure 5:**
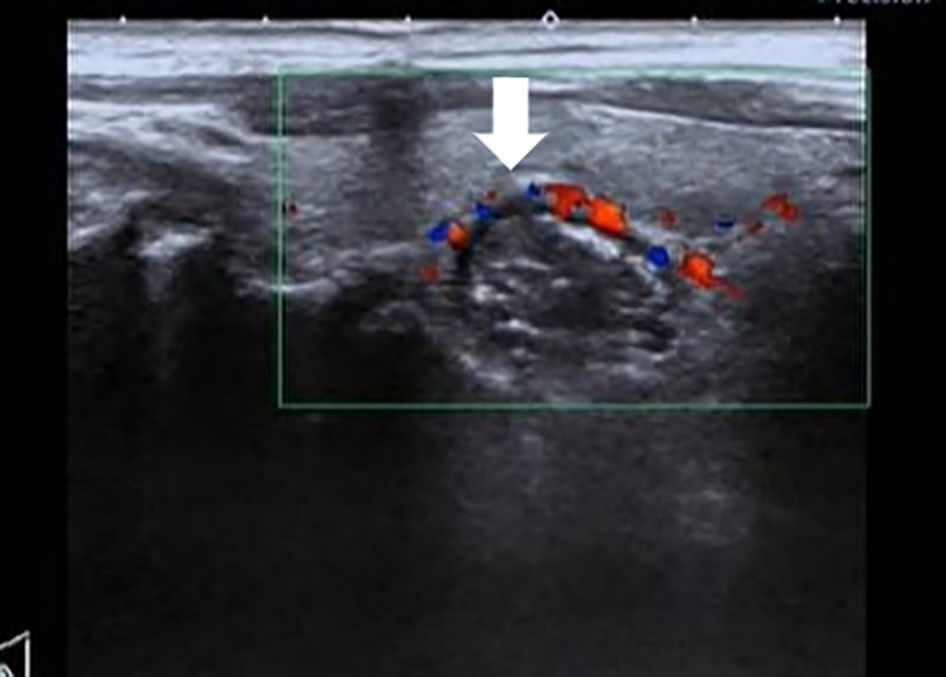
Punctate blood flow can be seen in the middle hypoechoic layer of the solid nodular diverticular (image from a 42-year-old woman).

Thirty patients were diagnosed with solid nodular diverticulum; the long diameter of all PEDs measured using sonography ranged from 9.1 mm to 58 mm (average 17.7 ± 12.1 mm). The diverticulum wall had a typical hyper-hypo-hyper-echogenic pattern (**Figure 3**). More people (n=24) exhibited “nodules” that protruded into the essence of the thyroid gland and were located behind the tegument at the thyroid posterior margin than did not (n=6). The high-low echogenic pattern intermixed with solid nodular diverticulum suggested striped strong internal echoes, with no acoustic tail or acoustic shadow. It could also be seen that the diverticulum wall and esophageal wall were continuous if multiple sections were visualized, such as with a crosscut and then cut-scanned (**Figure 4**). Punctate blood flow could be seen as a hypoechoic middle layer of the diverticulum wall (**Figure 5**).

#### Air-containing nodular diverticulum

**Figure 6:**
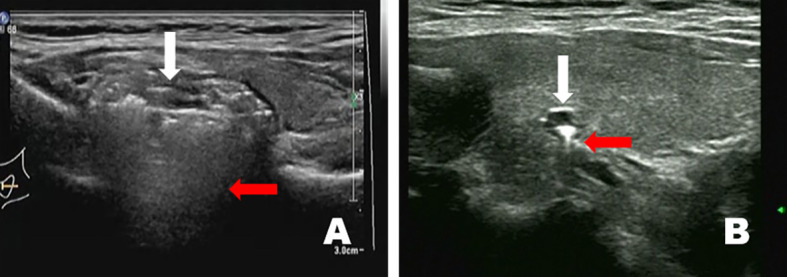
Some air-containing nodular diverticula are full of gas-like hyperechoicity internally and have a typical “V” tail sign **(A)**, image from a 35 year-old woman). Some have a hyperechoic linear appearance and sometimes have a typical “T” tail sign **(B)**, image from a 63-year-old woman).

Twenty-nine patients were diagnosed with air-containing nodular diverticulum; the long diameter of the PED measured using sonography ranged from 4.2 mm to 48 mm (average 11.2 ± 5.3 mm). Specific ultrasonographic features included gas-like strong internal echoes with acoustic tails. According to the amount of air inside, the diverticula were divided into the “rich-in” air-type and “single” air-type. The former was seen as a gas-like hyperechogenic signal inside the diverticulum, sometimes followed by a typical “V” tail sign (**Figure 6A**). The latter had a hyperechoic linear appearance in the diverticulum and was sometimes followed by a typical “T” tail sign (**Figure 6B**). The maximum diameters of the “T” and “V” acoustic tails were 1.2–2.2 cm and 2.5–4.8 cm respectively. The difference was statistically significant (p < 0.001).

#### Liquid-containing nodular diverticulum

Six patients were diagnosed with liquid-containing nodular diverticulum; the long diameter of all PEDs measured using sonography ranged from 4.5 mm to 32 mm (average 12.1 ± 4.7 mm). The main ultrasonic manifestations were characterized by a typical hyper-hypo-echoless sign from the outside to the inside, such that the diverticulum was echoless internally. Using dynamic scanning or after drinking water, the echoless cavity was visible on flow sense imaging; however, there was no obvious change in diverticulum size. The diverticulum wall and the esophageal wall were seen as continuous if cut scanned, and there was no blood flow visualized inside.

Atypical diverticular changes: Three patients had atypical diverticular changes. The long diameter of all PEDs measured using sonography ranged from 9.6 mm to 45 mm (average 16.4 ± 11 mm). There were regional convex parts outside the esophageal wall, with no obvious nodules. The convex segment continued with the esophageal wall and could be seen on slitting scanning. The inner diameter of the esophagus in the corresponding area increased.

## Discussion

ZD, the most common form of PED, results from herniation of the Killian triangle, which is an area of muscular weakness between the transverse cricopharyngeal fibers and oblique fibers of the thyropharyngeal muscle (9). Killian–Jamieson diverticulum is an uncommon condition, resulting from herniation of the Laimer membrane leading to a defect of the esophagus in the gullet (8). Common clinical presentations include dysphagia, regurgitation, choking, chronic cough, aspiration pneumonitis, globus, weight loss and, less commonly, dysphonia (10). For decades, the mainstay of treatment for ZD was an open surgical approach through a neck incision with performance of myotomy of the UES and removal or suspen-alternative and often preferable incisionless transoral approaches have been developed (11). With the development of minimally invasive endoscopic approaches for the esophagus in recent years, peroral endoscopic myotomy (POEM) in the treatment of esophageal diverticulum has been described recently in some reports due to its successful outcomes (12).

Most PEDs were located behind the left lobe of the thyroid gland. Due to the proximity of the thyroid gland, PED can occasionally mimic thyroid nodules and can be misdiagnosed as a thyroid mass (13). Currently, there are few reports on the sonographic findings of PEDs. In previous reports (7, 14–16), many researchers have misdiagnosed PED as thyroid nodules and performed FNA or surgical treatment.

We analyzed the ultrasonographic features in 68 cases of PED mimicking thyroid nodules and classified them into four types, aiming to improve the accuracy of preoperative diagnosis and to avoid misdiagnosis and inappropriate invasive treatment. Among them, solid nodular diverticulum was the most common type and can easily confused with thyroid nodules. The main reason for this is the absence of typical gas-like strong echogenicity, which is replaced by esophageal mucosa or food debris. Sometimes it is associated with a central hyperechoic area, which can be easily confused with thyroid nodules with microcalcifications. It is useful to diagnose and prevent patients from undergoing invasive procedures, such as aspiration and unnecessary surgery.

Nevertheless, based on the ultrasound features summarized in this study, the following characteristics of solid nodular diverticula can be used to distinguish them from true thyroid nodules if examined carefully. First, the diverticulum wall has a typical hyper (esophageal mucosa and debris)-hypo (esophageal myometrium)-hyper (esophageal serosa and posterior capsule of thyroid)-echogenic pattern. Second, the PED was found to be connected to the adjacent esophageal wall if multiple sections were scanned. Moreover, when the patient swallowed, the thyroid and diverticulum moved relative to each other. Eventually, real-time sonography was performed during the patient’s ingestion of water and demonstrated transient changes: an increase in the size of the lesion, a reduction in the definition of the margins, and heterogeneous echogenicity of the lesion’s contents. No apparent blood flow signal was observed in the lesions. However, thyroid nodules have marked hypoechogenicity with regular/irregular margins and extrathyroidal growth. Punctate blood flow can also be seen. It moves in the same direction as the thyroid during swallowing, but there was no significant change in the appearance of the lesion.

It is not difficult to diagnose air-containing nodular diverticula because there are obvious gas-like strong echoes. However, there are some atypical strong echoes, and acoustic tails need to be distinguished. Based on these changes, the diverticula were divided into “rich-in” gas-type followed by a typical “V” tail sign and “single” gas-type with a typical “T” tail sign. Because of the appearance of the acoustic tail, it is difficult to view the echo of the posterior structure of the diverticulum and its relationship with the esophageal wall. The instability of the gas causes the shape of the acoustic tail to easily change with movement, so that changes were more obvious with swallowing. Twenty-six patients were diagnosed with air-containing nodular diverticulum following X-ray barium meal examination, and three patients were diagnosed after a 3-year follow-up interview.

Therefore, PED should be considered if a nodular echo intensity is found behind the thyroid, accompanied by a gas-like stronger echo and an unstable acoustic tail. At this time, the patient should be asked to swallow to observe the relative movement between the lesion and thyroid gland or the patient should drink some water for real-time dynamic scanning to increase the accuracy of diagnosis.

The amount of liquid-containing nodular diverticula was relatively low in this study. The main ultrasonic manifestation was that the diverticulum cavity was echoless. We speculate that the cysts (fluid components) are related to the caudal direction of the diverticulum. If the caudal direction is directed to the centripetal end, the liquid or residue is easily deposited and difficult to remove. The echoless area may then be formed from the mucus secreted by the esophageal mucosa or the accumulation of liquid after drinking water. If the caudal directed to the head end, gases tend to accumulate more easily. The echoless area in the diverticulum can be scanned more clearly because the thyroid gland is a sound transmission window. However, this often puzzles ultrasonographers, leading to liquid-containing nodular diverticula to be diagnosed as lesions of parathyroid origin. PEDs with anechoic changes are very rare. Nine patients were considered to have PED, but the remaining three patients were considered to have parathyroid or lymphadenopathy. Six patients were finally diagnosed with PED following X-ray barium meal examination.

We reviewed the sonograms of these six patients and found that on real-time dynamic scanning, a slight floating could be visualized in the anechoic mass in the diverticulum cavity. Therefore, real-time dynamic scanning is necessary. In the case of ambiguous potential diverticula, it is suggested to use X-ray barium meal examination to aid diagnosis.

Atypical diverticular changes in this study refer to regional convex parts outside the esophageal wall with unfashioned nodules. The formation of PED is not only due to the weakness of the esophageal wall itself, but also due to an increase in pressure in the esophageal cavity. Strictly speaking, external traction can also lead the esophageal wall to form a diverticulum (15). Three patients showed atypical diverticulum changes in this study, and one patient had Hashimoto’s thyroiditis and a medical history of chronic bronchitis. Therefore, the diverticular changes may have been due to increased intraesophageal pressure. Meanwhile, inflammation can also pull the esophageal wall outward, resulting in segmental protrusion of the esophageal wall.

## Conclusion

PED mimicking thyroid nodules can occasionally be encountered in daily diagnostic work. Real-time dynamic and multi-slice scanning are essential to identifying solid nodular diverticula. Identifying the typical hyper-hypo-hyper echogenic pattern of the diverticulum wall is very important in the diagnosis. It is easier to diagnose when there is gas in the diverticulum; however, familiarity with the characteristic “V” and “T” tail can increase the confidence in diagnosis. The key to diagnosing liquid-containing nodular diverticulum is echogenicity in the diverticulum. Real-time sonography performed during the patient’s ingestion of water helps differentiate a PED from thyroid disease without the need for imaging with a contrast agent. For atypical diverticular changes, familiarity with the normal course of the esophageal wall is necessary to identify the disease. Awareness of changeable internal echoes and not mistaking strong echogenic foci caused by air for calcifications are the most important factors for making differentiating PEDs from thyroid nodules.

## Data availability statement

The original contributions presented in the study are included in the article/supplementary material. Further inquiries can be directed to the corresponding author.

## Ethics statement

The studies involving human participants were reviewed and approved by The First Hospital of China Medical University. Written informed consent for participation was not required for this study in accordance with the national legislation and the institutional requirements.

## Author contributions

ZB designed the study and wrote the manuscript; XW contributed to manuscript preparation and produce metadata; ZZ revised the work and approved the version to be published. All authors contributed to the article and approved the submitted version.

## References

[1] Marcy PY Benisvy D Poissonnet G Sadoul JL Thariat J. Zenker's diverticulum: The diagnostic power of ultrasound. Thyroid (2010) 20:1317–8. doi: 10.1089/thy.2010.0140 20932178

[2] Gray SL O'Neill G McGarry G. The predictive value of structured ultrasonographic staging for thyroid nodules. J Laryngol Otol (2014) 128:914–21. doi: 10.1017/S0022215114002072 25266276

[3] Walts AE Braunstein G. Fine-needle aspiration of a paraesophageal diverticulum masquerading as a thyroid nodule. Diagn Cytopathol (2006) 34:843–5. doi: 10.1002/dc.20570 17115442

[4] Ota K Onoe M Oka M Ota K Taniguchi K Sakaue M. Killian-jamieson diverticulum mimicking a thyroid nodule: A case report. J Gen Fam Med (2019) 20:62–4. doi: 10.1002/jgf2.222 PMC6399583 30873306

[5] Kwak JY Kim EK. Sonographic findings of zenker diverticula. J Ultrasound Med (2006) 25:639–42. doi: 10.7863/jum.2006.25.5.639 16632788

[6] Nauschuetz KK Ogden LL Starling CE Saleh MJ Golding AC Traweek ST. Pharyngoesophageal diverticula simulating thyroid nodules: An unusual occurrence with unique features. Diagn Cytopathol (2018) 46:193–7. doi: 10.1002/dc.23817 28925594

[7] Chen HC Chang KM Su WK. Incidental pharyngoesophageal diverticulum mistaken for a thyroid nodule: Report of two cases. Diagn Cytopathol (2019) 47:503–6. doi: 10.1002/dc.24144 30632292

[8] Abrams J. Sonographic differential diagnosis of thyroid nodule. pharyngoesophageal diverticulum. HNO (2011) 59:1215–8. doi: 10.1007/s00106-011-2314-z 21509615

[9] Siddiq MA Sood S Strachan D. Pharyngeal pouch (Zenker's diverticulum). Postgrad Med J (2001) 77:506–11. doi: 10.1136/pmj.77.910.506 PMC1742115 11470929

[10] Martinez Paredes JF Alfakir R Kasperbauer JL Rutt A. Zen. zenker diverticulum: Does size correlate with preoperative symptoms? Int Arch Otorhinolaryngol (2021) 26: e334–8. doi: 10.1055/s-0041-1730457 PMC9282963 35846818

[11] Castaneda D Azar FF Hussain I Lara LF Pimentel RR Alemar G. A cooperative approach for treatment of zenker's diverticulum. Surg Endosc (2022) 6:36. doi: 10.1007/s00464-021-08736-z 34524532

[12] Zeng X Bai S Zhang Y Ye L Yuan X Hu B. Peroral endoscopic myotomy for the treatment of esophageal diverticulum: An experience in China. Surg Endosc (2021) 35:1076–89. doi: 10.1007/s00464-020-07593-6 32347387

[13] Haddad N Agarwal P Levi JR Tracy JC Tracy LF. Presentation and management of killian jamieson diverticulum: A comprehensive literature review. Ann Otol Rhinol Laryngol (2020) 129:394–400. doi: 10.1177/0003489419887403 31707793

[14] Little RE Bock JM. Pharyngoesophageal diverticuli: Diagnosis, incidence and management. Curr Opin Otolaryngol Head Neck Surg (2016) 24:500–4. doi: 10.1097/MOO.0000000000000309 27636983

[15] Shanker BA Davidov T Young J Chang EI Trooskin SZ. Zenker's diverticulum presenting as a thyroid nodule. Thyroid (2010) 20:439–40. doi: 10.1089/thy.2009.0177 20373989

[16] Oertel YC Khedmati F Bernanke AD. Esophageal diverticulum presenting as a thyroid nodule and diagnosed on fine-needle aspiration. Thyroid (2009) 19:1121–3. doi: 10.1089/thy.2009.0136 19772399

